# Telomerase Complex from Yeast Saccharomyces cerevisiae Contains a Biotinylated Component

**Published:** 2009-07

**Authors:** D.M. Shcherbakova, M.I. Zvereva, O.A. Dontsova

**Affiliations:** 1 Department of Chemistry, Moscow State University

## Abstract

Telomerase adds telomeric repeats to single-stranded DNA at the ends of the chromosomes. This enzyme is a ribonucleoprotein complex. Telomerase from yeast Saccharomyces cerevisiae consists of TLC1 RNA, which serves as a template for the synthesis of telomeric repeats, telomerase reverse transcriptase Est2p, and a number of accessory proteins (Est1p, Est3p, Ku70/Ku80, and Sm-complex). We found that the yeast telomerase complex contains a biotinylated component. The telomerase fraction containing biotinylated protein is active in vitro and constitutes a small part of the total amount of active telomerase isolated from cells. We speculate about the nature of the biotinylated component.

## INTRODUCTION

Telomeres are located at the ends of eukaryotic chromosomes. These DNA-protein structures protect chromosomes from degradation and end-to-end fusion [1]. Telomerase is the enzyme that maintains the length of telomeres by adding telomeric DNA repeats to the 3≤-ends of chromosomal DNA [2]. Telomerase is active in the cells of organisms able to unlimitedly propagate, in 85% of cancers [3], and in cells of unicellular eukariotes (ciliates and yeasts) [2, 4].

Telomerase is a ribonucleoprotein complex [5, 6]. In yeast Saccharomyces cerevisiae, telomerase is composed of telomerase reverse transcriptase Est2p [7]; telomerase RNA TLC1 [8], a region of which serves as a template for telomeric repeats synthesis; accessory proteins Est1p [9] and Est3p [10] (without which telomerase is inactive in vivo) [11]; and several other proteins (Sm-proteins [12], Ku-proteins [13], etc.). Other than subunits of the telomerase complex, there are several proteins that interact with telomerase and contribute to telomerase functioning. For example, Cdc13p, which tethers telomerase to telomere and is crucial for its activity, is not a subunit of telomerase, although it interacts with Est1p [14]. Currently, a large number of genes whose absence leads to telomere shortening are known [15]. Some proteins (e.g., chaperone Hsp82p [16]) have also been shown to interact with subunits of the telomerase complex. Such proteins are probably important for complex assembly or form a transient part of telomerase on a particular step of regulation. Therefore, the sophisticated arrangement of telomerase is related to the complex regulation of its activity. It is known that telomere elongation occurs during the late S phase of the cell cycle [17, 18], and telomerase preferentially extends the shortest telomeric ends [19-21]. Also, regulation may occur at the assembly stage of the telomerase complex and the degradation of its components. Revealing new interactions between various proteins and telomerase subunits, post-translational modifications of proteins important for telomerase activity, and the elucidation of its roles will lead us to a better understanding of how telomerase functions in a cell.

We found that an active telomerase complex contains a biotinylated compound. Such telomerase complexes comprise less than half of the total amount of telomerase isolated from cells.

## Materials and methods 

### Strains 

Strain DBY-746 ≤ (ura3-52, leu2-3,112, trp1-289, his3-≤1) was used.

### Telomerase purification using streptavidi -based affinity chromatography 

The cell culture was grown to A600 = 1 in 3.2 liters of a SC≤- Trp medium supplemented with glucose. Cells were harvested by centrifugation (5 min, 5,000 rpm, 4°C, JA-10 rotor; Beckman, USA); washed four times with water; and, finally, washed with a "str" buffer (20 mM Tris-HCl, pH 7.5, 100 mM NaCl, 2 mM MgCl2, 1 mM dithiothreitol (DTT ), 0.1 mM EDTA, 10% (v/v) glycerol, 0.1% (v/v) Triton X-100). The cell pellet was mechanically disrupted in liquid nitrogen and thawed on ice with 10 ml of an ice-cold "str" buffer supplied with "Complete protease inhibitor" (Roche, Switzerland), phenylmethylsulfonyl fluoride (0.5 mM), and RN asin (40 U/ml of extract) (Helicon, Russia). The cell debris was removed by centrifugation (once at 5 min, 5,000 g and twice at 15 min, 15,000 rpm; 4°C, JA-20 rotor, Beckman, USA). An aliquot of the extract was used to measure the total protein concentration using the Compat-Able Protein Assay Kit and BCA Protein Assay Reagent (Pierce Biotechnology, USA). During the experiment, the extract was pretreated with avidin: 10 ≤g of avidin per 1 mg of total protein was added, and the sample was incubated for 10 min at 4°C. Then, the yeast extract (10 mg/ml total protein) was added to a streptavidin-sepharose resin preequilibrated with a "str" buffer (200 ≤l of resin from GE Healthcare (USA) for 10 ml of extract). The mixture was incubated at 4°C for 1.5 h under agitation. Then, the resin was washed six times with a "str" buffer. The resin that was obtained and all other fractions were frozen in liquid nitrogen and stored at - 80°C for further analysis.

### Telomerase purification using DEAE-based anionic chromatography 

Yeast telomerase was prepared according to [4, 22] with a slight modification: the telomerase fraction was obtained by elution from a DEAE-cellulose column with a linear gradient concentration of sodium acetate (from 100 mM to 1 M). From 10 ml of the initial extract (10 mg/ml total protein), 1ml of the active telomerase fraction was obtained. Then it was aliquoted, frozen in liquid nitrogen, and stored at - 80°C for further analysis.

### Fractionation of S100 extract and DEAE-fraction in a glycerol density gradient 

The yeast extract was prepared as described above for the isolation of telomerase using chromatography on straptavidin-sepharose, except that the YPD medium was used for cell growth and a lysis buffer (25 mM Tris-HCl (pH 7.5), 300 mM NaOAc, 2 mM MgCl2, 1 mM DTT , 0,1 mM EDTA, 10% (v/v) glycerol) was used instead of a "str" buffer. Then, the S100 extract was obtained by the ultracentrifugation of the yeast extract (1h, 100,000g, 4°C, Ti-70 rotor; Beckman, USA). Then, it was concentrated on a Vivaspin 20 (Sartorius, Germany). The S100 extract (0.5 ml, 15 mg/ml total protein) or DEAE-fraction (0.5 ml) was loaded onto a 15-40% glycerol gradient (10.5 ml, in a lysis buffer). Ultracentrifugation was performed at the following conditions: 24h, 40,000rpm, 4°C, SW41 rotor; Beckman. Twentytwo fractions (0.5 ml each) were gathered, frozen in liquid nitrogen, and stored at - 80°C for further analysis.

### In vitro telomerase assay 

Ten microliters of the sample (obtained after isolating the streptevidin-sepharose suspension, DEAE-fraction, or the fraction obtained by ultracentrifugation) was used in the elongation reaction. The final reaction mixture (20 ≤l) contained 50 mM Tris-HCl (pH 8.0), 5 mM MgCl2, 1 mM DTT , 1 mM spermidine, 0.05 mM EDTA (contributed by telomerase fraction), 5% (v/v) glycerol or more for the sample obtained in ultracentrifugation (contributed by telomerase fraction), 100 ≤M dTT P, 20 ≤Ci [≤-32P]dGTP (3000 Ci/mmol), and 5 ≤M oligodeoxyribonucleotide TE L11 (5≤-TGGTGTGTGGG-3≤). Control reactions were pretreated with RN ase A (1 ≤l of 10 mg/ml solution, 30 min, 30°C). The telomerase reaction was carried out for 1 h at 30°C followed by the addition of 200 ≤l of a "stop" buffer (20 mM Tris_HCl, pH 8.0, 20 mM EDTA, 0.2% SDS) and 3 ≤l proteinase K (20 mg/ml). After incubation at 30°C for 1 h, the reaction products were extracted twice with equal volumes of phenol, once with an equal volume of chloroform-isoamyl alcohol (24 : 1), and precipitated with 3 volumes of ethanol in the presence of 1/10 volume of 3 M NaOAc and 5 ≤g of tRNA from E. coli as a carrier. The pellet was washed twice with 80% ethanol, dried and dissolved in the formamide loading buffer (80% of deionized formamide, 1 ≤ TBE buffer, 0.1% xylene cyanole, and 0.1% bromophenol blue). Reaction products, along with 5≤-[32P]-phosphorylated oligodeoxyribonucleotide TE L11 as a length marker, were separated electrophoretically in 15% TBE denaturing PAAG. The gel was dried and analyzed using the PhosphorImager system (Molecular Dynamics, USA).

### Western bloting detection of biotinylated proteins

In the samples obtained after ultracentrifugation, the proteins were precipitated by the slow addition of 5 volumes of ice-cold acetone and incubation of the mixture for 24 h at - 20°C. The pellet was washed with ice-cold acetone, dried, dissolved in a buffer containing 8M Urea and 70 mM Tris-HCl (pH 7.5), and diluted in water. Protein mixtures were separated by electrophoresis in 15% SDS-PAGE according to Laemmli [23]. Proteins from extract and DEAE-fraction were separated in 10-12% SDS-PAGE without the precipitation step. They were transferred to nitrocellulose (GE Healthcare) or PVDF (BioRad) membranes. To detect biotinylated proteins, streptavidin-HRP conjugate [24] and ECL kit (GE Healthcare) were used.

### RT-PCR analysis 

RNA for RT -PCR was obtained using phenol extraction, chloroform-isoamyl alcohol (24 : 1) extraction, and ethanol precipitation in the presence of 1/10 volume 3 M NaOAc and tRNA from Escherichia coli as a carrier (5 ≤g per probe). Samples containing RNA bound to the streptavidin-sepharose resin were pretreated with proteinase K as described above for telomerase assay in vitro. All the samples were treated with DNase I (1 U/≤g nucleic acids or 1 U/100 ≤l fraction obtained by ultracentrifugation, 1 h at 37°C). After that, RNA was purified from proteins with the use of extraction and precipitation as described above. All the samples were diluted in an equal amount of water (usually 10 ≤l), and RNA concentration was measured spectrophotometrically at 260 nm. The volume of the initial extract containing 0.1-0.5 ≤g RNA was used for RT-PCR analysis. An equal volume of unbound fraction and corresponding volume of bound fraction corrected for TLC1 concentration in binding from the extract were taken for RT-PCR analysis. If fractions obtained by ultracentrifugation were analyzed, 1 ≤l of the sample was used for analysis. For RT-PCR reactions, a OneStep RT-PCR Kit (Qiagen) was used. The gene-specific primers for TLC1 were P2 (5≤-GTTATTCTAGTTC G-3≤) and T8 (5≤-CGAAGGCATTAGGAGAAG-3≤). RT -PCR products were analyzed by electrophoresis in 2% agarose gel with a TBE buffer (89 mM Tris, 89 mM boric acid, 2 mM EDTA, pH 8.3).

## Results and discussion 

### The active yeast telomerase complex contains biotinylated protein 

**Fig. 1.**
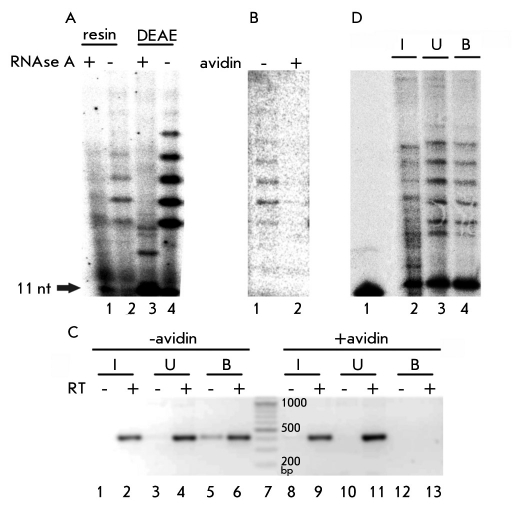
Isolation of yeast telomerase using chromatography on streptavidin-sepharose . (a) Activity assay (elongation of oligodeoxyribonucleotide TEL11) of telomerase isolated on streptavidin-sepharose from extract. RNase A(+) and RNase A(-) are the reactions with and without pretreatment with RNase A. (1, 2) the products of TEL11 elongation by telomerase isolated on streptavidin-sepharose; (3, 4) the same for telomerase isolated on DEAE-cellulose. (b) The same as in (a) with pretreatment with and without avidin. Avidin(+) and Avidin(-) are the reactions with and without pretreatment with avidin. (1, 2) the same as (a; 1, 2). (c) RT-PCR analysis of TLC1 RNA in samples obtained by telomerase isolation on streptavidin-sepharose with and without pretreatment with avidin. RT(-) and RT(+) are RT-PCR analyses without and with reverse transcription reaction. (1, 2) RT-PCR products obtained in an analysis of the initial extract (I); (3, 4) the same for unbound fraction (U); (5, 6) the same for bound fraction (B) without avidin pretreatment; (7) molecular weight marker; (8-13) the same as in (8-13) with avidin pretreatment. (d) Activity assay of telomerase isolated on streptavidinsepharose from DEAE-fraction. (1) 5’-[32P]- phosphorylated oligodeoxynucleotide TEL11; (2) the products of TEL11 elongation by telomerase of initial DEAE-fraction (I); (3) the same for unbound fraction (U); (4) the same for bound fraction (B), taken in a 4-times excess

It is known that yeast telomerase activity cannot be detected directly in the yeast extract obtained by the disruption of yeast cells. This becomes possible only after the step of enriching the telomerase complex by specifically binding the complex subunits on affinity resin (Est1 [25], Est2p [26], Est3p [10]) or by enriching the whole complex using anion-exchange chromatography [4, 22]. We found that when the yeast extract was bound to streptavidin-sepharose, active telomerase could be detected on the resin (Fig. 1a). This fact indicates that active telomerase is concentrated on affinity resin because there is no activity in the initial extract. The pattern of detected activity is the same as the pattern of activity of telomerase isolated via the anion-exchange chromatography on DEAE-cellulose and corresponds to the addition of one telomeric repeat in the in vitro reaction (Fig. 1a).

To test if the binding of telomerase with streptavidin-Sepharose results from the "biotin-streptavidin" interaction (dissociation constant Kd= 10-14 M [27]), we performed the binding experiment with the pretreatment of the yeast extract with avidin. Adding avidin is a common way to prevent binding with the streptavisin-sepharose of biotinylated proteins from the yeast extract [28], because the "biotin-avidin" interaction (dissociation constant Kd= 10-15 M [29]) completely blocks the "biotin-sreptavidin" interaction. In fact, adding avidin prevents the binding of telomerase with the affinity resin (Fig. 1b). This result indicates that the interaction is specific. Telomerase binding to the resin and the prevention of this binding by adding avidin were also shown for TLC1 RNA by RT-PCR analysis (Fig. 1c).

In the next step, we performed the binding of telomerase that had already been purified via chromatography on DEAE-cellulose with the affinity resin. We found that active telomerase in fact binds with streptavidin-sepharose, but only partially (Fig. 1d). This could be due to the fact that only part of telomerase complexes contains biotinylated protein. Also, we could not exclude the possibility that, during purification on DEAE-cellulose, some active telomerase complexes lose their biotinylated component.

### The biotinylated protein-telomerase subunit has an apparent mass of 50 kDa and it is a constituent of only part of the total amount of active telomerase 

**Fig. 2.**
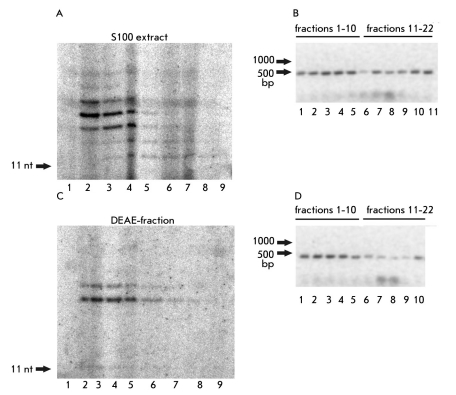
Analysis of fractions obtained by ultracentrifugation in a glycerol density gradient (22 fractions from one gradient) . (a) Telomerase activity assay in fractions obtained by ultracentrifugation of S100 extract. (1-9) The products of TEL11 elongation by telomerase in fractions 1-9, respectively. (b) RT-PCR analysis of TLC1 RNA in fractions obtained by ultracentrifugation of S100 extract. (1-9) RT-PCR products obtained in an analysis of fractions 1-22, respectively, two adjacent fractions in each lane (1) 1 and 2 fractions; (2) 3 and 4 fractions, etc.). (c) The same as in (a) for fractions obtained by ultracentrifugation of DEAE-fraction. (d) The same as in (b) for fractions obtained by the ultracentrifugation of DEAE-fraction

**Fig. 3.**
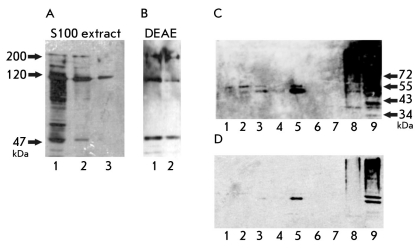
Western blotting analysis of biotinylated proteins in the initial S100 extract, DEAEfraction and fractions obtained by ultracentrifugation in glycerol density gradient . (a) Western blotting analysis of proteins in the initial S100 extract. (1-3) biotinylated proteins from extract (the amount of samples decreases from 1 to 3). (b) Western blotting analysis of proteins in DEAE-fraction. (1, 2) biotinylated proteins from DEAE-fraction in decreasing amounts (from 1 to 2). (c) Western blotting analysis of proteins in fractions obtained by the ultracentrifugation of DEAE-fraction. (1-8) biotinylated proteins from fractions 1-8, respectively; (9) biotinylated proteins from initial DEAE-fraction. (d) The same as in (c) with the quick exposure of a membrane to a film

We fractionated the yeast S100 extract as well as telomerase purified on DEAE-cellulose in a glycerol density gradient using ultracentrifugation. Centrifugation of both types of samples was done simultaneously and under the same conditions. It is known that yeast telomerase sediments as 19S when ultracentrifugated [30]. We tested the obtained fractions for the presence of TLC1 RNA, telomerase activity, and biotinylated proteins (Figs. 2, 3). The distribution of active telomerase throughout the fractions is shown on Figs. 2a and 2c, and the distribution of TLC1 RNA is shown on Figs. 2b and 2d.

We used Western blotting for the detection of biotinylated proteins in the initial yeast extract (Fig. 3a) and initial DEAEfraction (Fig. 3b), as well as the distribution of biotinylated proteins throughout the fractions obtained by ultracentrifugation (Figs. 3c, 3d).

The three most intense bands corresponding to 47, 120, and 200 kDa are readily detected in the extract and DEAEfraction (Figs. 3a, 3b). There are only a few known biotinylated proteins in yeast: Acc1p (250 kDa [31]), Hfa1p (242 kDa [32]), Pyc1p (130 kDa [33]), Pyc2p (130 kDa [33]), Dur1,2p (202 kDa [34]), and Arc1p (42 kDa [35]). In general, the detected bands correspond to those described in the literature [35] and anticipated in accordance with the molecular masses of the known proteins.

In the case of the fractionation of telomerase enriched on DEAE-cellulose, one could see that biotinylated protein with an apparent molecular mass of 50 kDa comigrates and enriches with the telomerase complex. This is obvious from comparing Figs. 2c and 3c. In the case of S100 extract fractionation, we could not find a correlation between the presence of telomerase activity and the presence of biotinylated protein, because a series of bands corresponding to all yeast biotinylated proteins were detected in all fractions like in the initial extract (data not shown).

We have shown that the yeast telomerase complex contains biotinylated protein. We have not managed to establish what protein it is and what its function is. Our attempts to use MALDI-TOF analysis for identifying the protein of interest were unsuccessful due to a small amount of telomerase per cell (approximately 29 molecules of TLC1 RNA per haploid yeast cell [36]) and the fact that only a portion of the total amount of active telomerase contains biotinylated protein. Also, it should be noted that the elution of proteins bound on streptavidin-sepharose by the "biotin-streptavidin" interaction is a difficult task, because, due to the strength of the interaction, it is not quantitative [37].

**Fig. 4.**
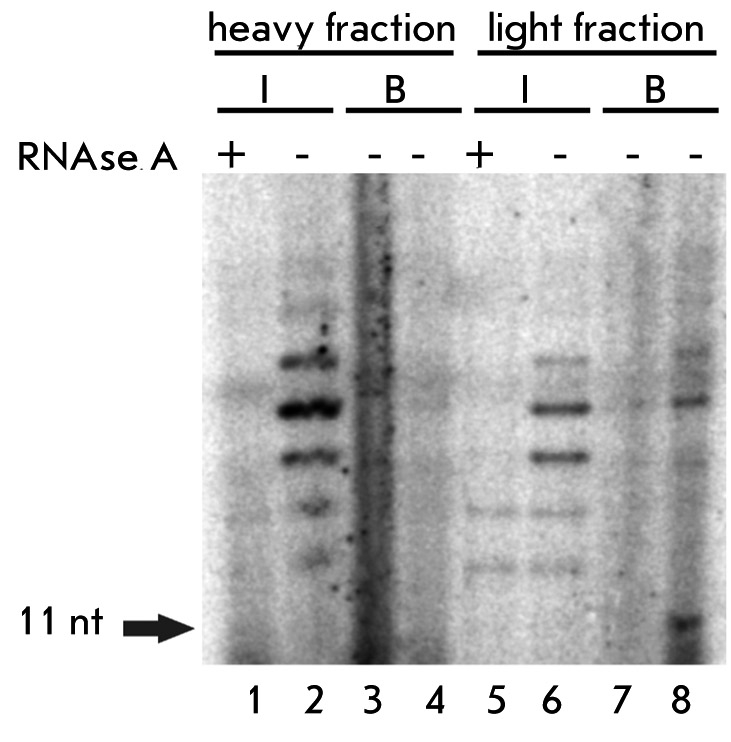
Binding of heavy (2 and 3 fractions) and light (4 and 5 fractions) telomerase complexes obtained by S100 extract ultracentrifugation in glycerol gradient, with streptavidin-sepharose. RNase A(+) and RNase A(-) are the reactions with and without pretreatment with RNase A. (1, 2) the products of TEL11 elongation obtained by the telomerase of the initial heavy fraction (I); (3, 4) the same for bound fraction (B), taken in a 4-times excess and 10-times excess, respectively; (5-8) the same as in (1-4) for light fraction

It is obvious from Figs. 2c and 3c that the detected biotinylated protein comigrates with the lighter portion of telomerase complexes. An interesting proposition could be made on the basis of this fact and the fact that biotinylated protein is present in less than half of the active telomerase complexes isolated on DEAE-cellulose. We speculated that the biotinylated protein is a constituent of only the light telomerase complexes that may be in the maturation process but already active in vitro. We tested this idea for fractions obtained by the ultracentrifugation of the S100 extract. We combined two heavy fractions, and the same was done for two light fractions. Then, we performed the binding of these two samples with streptavidin-sepharose. In fact, one could see that the active telomerase from the heavy fractions does not bind to streptavidin-sepharose, while telomerase from light fractions binds (Fig. 4). So our proposition is proved, and this result disproves the hypothesis that the biotinylated component dissociates during purification on DEAE-cellulose, because, even without this purification step, only a portion of telomerase complexes contains biotinylated protein.

Our data (the molecular mass of a candidate protein is about 50 kDa) and the data existing in the literature allow us to speculate about the nature of biotinylated protein and its function. As was already noted, there are only a few known biotinylated proteins in yeast. These are three types of carboxylases containing biotin as a cofactor: acetyl-CoA carboxylases Acc1p (250 kDa [31]), Hfa1p (242 kDa [32]); piruvate carboxylases Pyc1p (130 kDa [33]), Pyc2p (130 kDa [33]); urea amidolyase Dur1,2p (202 kDa [34]; and protein Arc1p (42 kDa [35]), a cofactor of aminoacyl-tRNA synthetase [38]. Arc1p is also known to bind quadruplex DNA [39]. Of these proteins, only Arc1p is the most similar in molecular mass to the discovered biotinylated protein. It is interesting that Arc1p does not contain a canonical sequence for biotinylation by biotin-protein ligase. Moreover, biotinylation is not functionally important for Arc1p activity [35]. As an RNA-binding protein and a protein that binds quadruplex DNA, Arc1p seems to be a possible candidate for the biotinylated component of telomerase. It is also possible that telomerase contains a protein that is not already known to be biotinylated, interacting as Arc1p with biotin ligase Bpl1p and as Arc1p without a canonical sequence for biotinylation.

An interesting question arises as to the role of biotinylated protein in the telomerase complex. We have found that only the lighter portion of telomerase complexes contains biotinylated protein. It is known that the main components of telomerase crucial for the elongation of telomeric DNA are telomerase reverse transcriptase Est2p and telomerase RNA TLC1 [5]. Other components are necessary for regulation, assembly, biogenesis and degradation of the complex [5]. Some of them join telomerase only transiently at a particular moment in a cell cycle. For example, proteins Est1p and Est3p, crucial for telomerase activity in vivo, become part of the complex only in the late S/G2 phase of the cell cycle [18]. Our data indicate that biotinylated protein is not a permanent component of telomerase complex. It joins telomerase only transiently, on a particular step of assembly, biogenesis, regulation or degradation and probably participates in these processes.

## Acknowledgements

This work was supported by the Russian Foundation for Basic Research (grants 08-04-01220-a and 07-04-92119-a) and State contract (grant P800).
